# Theoretical investigation of dynamics and concurrence of entangled ${{\mathcal {P}}}{{\mathcal {T}}}$ and anti-${{\mathcal {P}}}{{\mathcal {T}}}$ symmetric polarized photons

**DOI:** 10.1038/s41598-023-34516-x

**Published:** 2023-05-26

**Authors:** Javed Akram, Chao Zheng

**Affiliations:** 1 eleQtron GmbH, Martinshardt 19, 57074 Siegen, Germany; 2 Department of Physics, COMSATS University Islamabad, Islamabad, 45550 Pakistan; 3 Department of Physics, College of Science, North China University of Technology, Beijing, 100144 China

**Keywords:** Single photons and quantum effects, Quantum information, Theoretical physics, Quantum optics

## Abstract

Non-Hermitian systems with parity-time $\mathcal {(PT)}$ symmetry and anti-parity-time $\mathcal {(APT)}$ symmetry have exceptional points (EPs) resulting from eigenvector co-coalescence with exceptional properties. In the quantum and classical domains, higher-order EPs for ${{\mathcal {P}}}{{\mathcal {T}}}$ symmetry and $\mathcal {APT}$-symmetry systems have been proposed and realized. Both two-qubits $\mathcal {APT}$-$\mathcal {APT}$ and ${{\mathcal {P}}}{{\mathcal {T}}}$-${{\mathcal {P}}}{{\mathcal {T}}}$ symmetric systems have seen an increase in recent years, especially in the dynamics of quantum entanglement. However, to our knowledge, neither theoretical nor experimental investigations have been conducted for the dynamics of two-qubits entanglement in the ${{\mathcal {P}}}{{\mathcal {T}}}$-$\mathcal {APT}$ symmetric system. We investigate the ${{\mathcal {P}}}{{\mathcal {T}}}$-$\mathcal {APT}$ dynamics for the first time. Moreover, we examine the impact of different initial Bell-state conditions on entanglement dynamics in ${{\mathcal {P}}}{{\mathcal {T}}}$-${{\mathcal {P}}}{{\mathcal {T}}}$, $\mathcal {APT}$-$\mathcal {APT}$ and ${{\mathcal {P}}}{{\mathcal {T}}}$-$\mathcal {APT}$ symmetric systems. Additionally, we conduct a comparative study of entanglement dynamics in the ${{\mathcal {P}}}{{\mathcal {T}}}$-${{\mathcal {P}}}{{\mathcal {T}}}$ symmetrical system, $\mathcal {APT}$-$\mathcal {APT}$ symmetrical system, and ${{\mathcal {P}}}{{\mathcal {T}}}$-$\mathcal {APT}$ symmetrical systems in order to learn more about non-Hermitian quantum systems and their environments. Entangled qubits evolve in a ${{\mathcal {P}}}{{\mathcal {T}}}$-$\mathcal {APT}$ symmetric unbroken regime, the entanglement oscillates with two different oscillation frequencies, and the entanglement is well preserved for a long period of time for the case when non-Hermitian parts of both qubits are taken quite away from the exceptional points.

## Introduction

The discovery of parity-time-symmetric Hamiltonians in 1998 attracted significant attention to this new class of non-Hermitian Hamiltonians^1^. Multiple physical systems have been found to have real eigenvalues associated with ${{\mathcal {P}}}{{\mathcal {T}}}$-symmetric Hamiltonians^2–6^. Although this ${{\mathcal {P}}}{{\mathcal {T}}}$ symmetry is spontaneously broken at some points and the intended eigenvalues may vanish, as the eigenvalues become complex at those points^7^. There have been numerous theoretical and experimental investigations of ${{\mathcal {P}}}{{\mathcal {T}}}$ symmetry in non-Hermitian systems both classical and quantum mechanical^8–25^. ${{\mathcal {P}}}{{\mathcal {T}}}$-symmetric systems have also been explored for many remarkable quantum phenomena, including the existence of critical phenomena^26^, the increase in entanglement^27^, the transfer of chiral populations^28^, the decoherence dynamics^29^, and the retrieval and criticality of the information^30–32^. A number of theoretical studies have investigated how entanglement disappears suddenly in ${{\mathcal {P}}}{{\mathcal {T}}}$-symmetric systems^33–35^, while^36^ has addressed effective entanglement recovery via operators. ${{\mathcal {P}}}{{\mathcal {T}}}$-symmetric systems have been reported to exhibit entanglement, precision metrology, and enhanced sensing capabilities^37–41^. The ${{\mathcal {P}}}{{\mathcal {T}}}$-symmetric quantum walk is based on topological edge states^42^, the broken ${{\mathcal {P}}}{{\mathcal {T}}}$ symmetry is stable entropy states^43^, and optomechanical dynamics can be demonstrated under both regimes^44^. In contrast, there has recently been considerable interest in another important counterpart called anti-parity-time $\mathcal {(APT)}$ symmetry.

**Figure 1:**
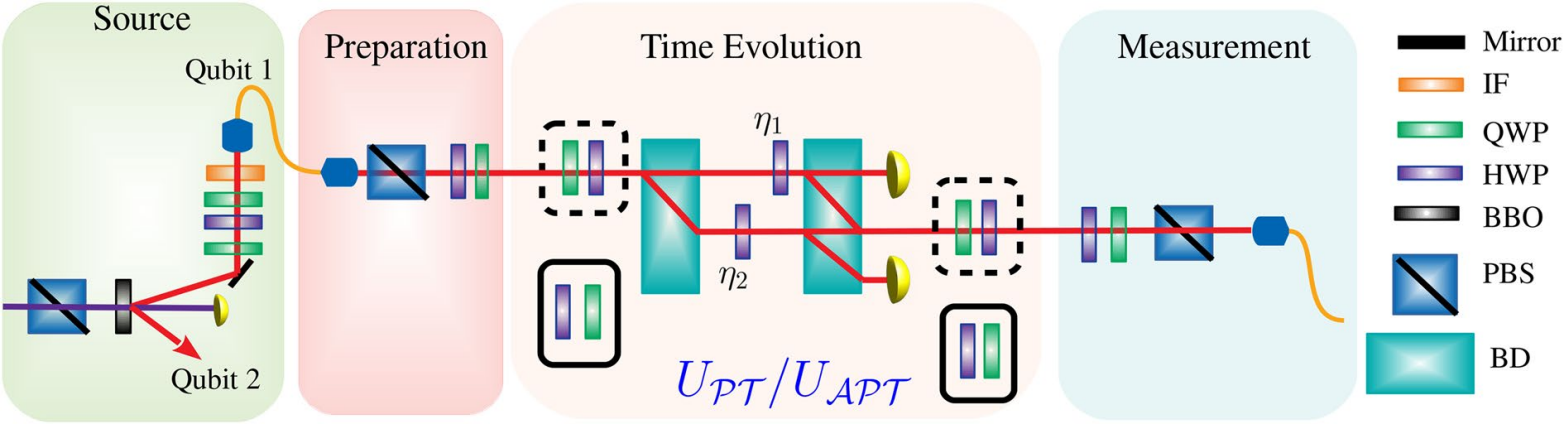
Experimental mechanism. Green area: A 404 nm laser light is passed through a type-I spontaneous parametric down-conversion using a nonlinear barium-borate crystal to generate pairs of 808 nm single photons. Red area: A signal photon exists in a linear polarization state after passing through a 3 nm interference filter (IF). Brick area: Beam displacement devices (BDs) are used in conjunction with half-wave and quarter-wave plates (HWPs and QWPs) in order to construct ${\hat{U}}_{j,{{\mathcal {P}}}{{\mathcal {T}}}}$ and ${\hat{U}}_{j,\mathcal {APT}}$. Quantum-state tomography is used in the final measurement part to construct the density matrix. Here PBS stands for a polarization beam splitter. Simulating ${\hat{U}}_{j,{{\mathcal {P}}}{{\mathcal {T}}}}$ is accomplished by choosing the plate combinations in the dotted black wireframe while simulating ${\hat{U}}_{j,\mathcal {APT}}$ by selecting the plates in the solid black wireframe, for more detail please follow^45^.

The Hamiltonians for the ${{\mathcal {P}}}{{\mathcal {T}}}$ and $\mathcal {APT}$ systems have a one-to-one correspondence, i.e., the Hamiltonian $H_{j,{{\mathcal {P}}}{{\mathcal {T}}}}$ in a ${{\mathcal {P}}}{{\mathcal {T}}}$-symmetric system has a counterpart $H_{j,\mathcal {APT}}$ in an $\mathcal {APT}$-symmetric system. Despite only being different by an imaginary number, $\mathcal {APT}$-symmetric systems exhibit quite different dynamic characteristics than ${{\mathcal {P}}}{{\mathcal {T}}}$-symmetric ones. An $\mathcal {APT}$-symmetric system has recently been studied by a number of research groups^46–48^. Choi et al.^49^ have shown that ${{\mathcal {P}}}{{\mathcal {T}}}$-symmetric systems exhibit distinct symmetry during ${{\mathcal {P}}}{{\mathcal {T}}}$ operations, whereas $\mathcal {APT}$-symmetric systems do not exhibit the same symmetry. For a full understanding of open quantum systems, it is important and interesting to study the dynamic properties of $\mathcal {APT}$-symmetric systems^50–53^. Recently^48,54^, it was shown there are exceptional points when real eigenvalues change from symmetric unbroken phase to symmetric broken phase for $\mathcal {APT}$ systems. Furthermore, optical materials with balanced positive and negative indices have been observed in symmetric $\mathcal {APT}$ systems^50^ and with constant refraction in optical systems^51^. Experiments in optics have demonstrated $\mathcal {APT}$-symmetric systems^52,55,56^, in atoms^57^, in electrical circuit resonators^49^, in diffusive systems^47^, and in waveguides^56^. Further, Ref.^54^ showed that $\mathcal {APT}$ symmetry could be spontaneously broken by spinning a lossy resonator in a linear device. A warm atomic-vapor cell was used in reference^58^ to demonstrate optical $\mathcal {APT}$ symmetry for the first time. Several experiments^59,60^ demonstrated dynamic encirclement of exceptional points. $\mathcal {APT}$- and ${{\mathcal {P}}}{{\mathcal {T}}}$-symmetric systems were experimentally demonstrated in reference^45^. To our knowledge, neither theoretical nor experimental investigations have been conducted on the dynamics of entanglement during the ${{\mathcal {P}}}{{\mathcal {T}}}$-$\mathcal {APT}$ symmetric regime. Moreover, we investigate the impact of different initial Bell-state conditions on entanglement dynamics. Additionally, the entanglement dynamics of the ${{\mathcal {P}}}{{\mathcal {T}}}$-${{\mathcal {P}}}{{\mathcal {T}}}$ symmetric system, $\mathcal {APT}$-$\mathcal {APT}$ symmetric system, and ${{\mathcal {P}}}{{\mathcal {T}}}$-$\mathcal {APT}$ symmetric system are also analyzed, as they reveal different phenomena from Hermitian quantum systems as well as their relationship to their environments.

Our analysis in “Experimental setup and methodology” section provides a brief introduction to the physical coupling of qubits as well as a Hamiltonian for the physical coupling. In this section, we also introduce the coupling of the two-qubits ${{\mathcal {P}}}{{\mathcal {T}}}$-${{\mathcal {P}}}{{\mathcal {T}}}$ symmetric system, $\mathcal {APT}$-$\mathcal {APT}$ symmetric system, and ${{\mathcal {P}}}{{\mathcal {T}}}$-$\mathcal {APT}$ symmetric systems. We also describe a brief experimental procedure as given in Fig.  1. Initial states can be achieved, by using a type-II phase-matched nonlinear barium-borate (BBO) crystal of 0.4 mm thick, and optical axes perpendicular to each other pump with a 404 nm pump laser (130 mW) to generate the Bell state through a degenerate spontaneous parametric down-conversion. Wave plates are used to calibrate quantum state (half-wave plate sandwiched between quarter-wave plates) as shown in Fig. 1. We examine the entanglement for identical experimental construction scenarios i.e., the ${{\mathcal {P}}}{{\mathcal {T}}}$-${{\mathcal {P}}}{{\mathcal {T}}}$ symmetric system, and the $\mathcal {APT}$-$\mathcal {APT}$ symmetric system, in “Identical experimental construction for both qubits” section. We study the dynamics of the entangled photon in different experimental construction for both qubits i.e., ${{\mathcal {P}}}{{\mathcal {T}}}$-$\mathcal {APT}$ symmetric system in “[Sec Sec6]” section. Our findings and conclusions are summarized in “Summary and conclusion” section.

## Experimental setup and methodology

In the case of a single qubit, a nontrivial Hamiltonian takes the form^61^

**Figure 2:**
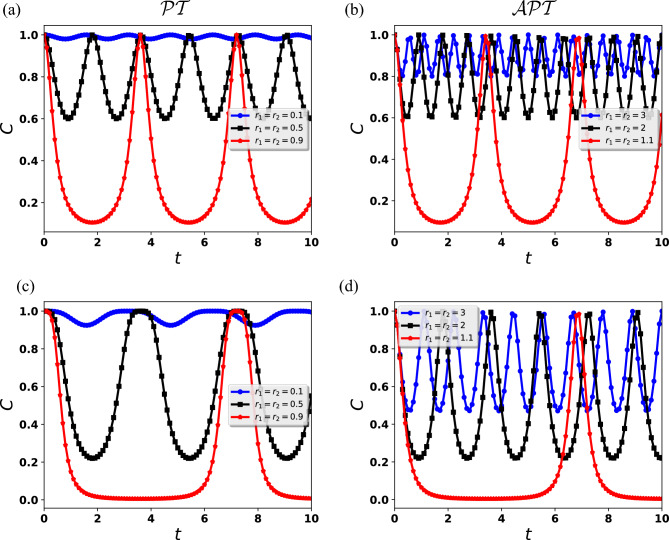
The dynamics of the concurrence for the two qubits initially in the Bell state ${|{B_{00}}\rangle } = \frac{1}{\sqrt{2}} \left( {|{00}\rangle } + {|{11}\rangle } \right)$ (**a**, **b**) and ${|{B_{01}}\rangle }= \frac{1}{\sqrt{2}} \left( {|{01}\rangle } + {|{10}\rangle } \right)$ (**c**, **d**). The time evolution of the concurrence in the ${{\mathcal {P}}}{{\mathcal {T}}}$-${{\mathcal {P}}}{{\mathcal {T}}}$ symmetric system is plotted in (**a**,** c**). The dynamics of concurrence in the $\mathcal {APT}$-$\mathcal {APT}$ symmetric system are shown in (**b**, ** d**). For simplicity, we took the values of parameters $r_1$ and $r_2$ equal for all cases.

1 $$\begin{aligned} {\hat{H}}_j= e^{i \psi _j} \left( s_j \sigma _x + i \gamma _j \sigma _z \right) = s_j e^{i \psi _j } \begin{pmatrix} i r_j &{} 1 \\ 1 &{} -i r_j \end{pmatrix}, \end{aligned}$$

here, $j={1,2}$ expresses qubit one and two, $\sigma _x$ and $\sigma _z$ are Pauli operators, $s_j >0$ defines energy scale parameter, $r_j =\frac{\gamma _j }{s_j }>0$ describes the degree of non-Hermiticity and $\psi _j$ depicts a ${{\mathcal {P}}}{{\mathcal {T}}}$-symmetric and $\mathcal {APT}$-symmetric Hamiltonian by taking $\psi _j =0$ and $\psi _j = \frac{\pi }{2}$, respectively. The eigenvalues of generalized Hamiltonian are calculated as

2 $$\begin{aligned} E_j =\pm s_j e^{i \psi _j } \sqrt{1-r_j^2}, \end{aligned}$$

for ${{\mathcal {P}}}{{\mathcal {T}}}$-symmetric ($\psi _j =0$) the eigenvalues are imaginary for $r_j >1$ (${{\mathcal {P}}}{{\mathcal {T}}}$-symmetric broken regime) and for $0<r<1$ (${{\mathcal {P}}}{{\mathcal {T}}}$-symmetric unbroken regime) eigenvalues are real. On the other hand, for $\mathcal {APT}$-symmetric ($\psi _j =\frac{\pi }{2}$) the eigenvalues are imaginary for $0<r_j <1$ ($\mathcal {APT}$-symmetric broken regime) and real for the case $r_j >1$ ($\mathcal {APT}$-symmetric unbroken regime). For both scenarios, eigenvalues get zero at exceptional point (EP) $r_j =1$. As $s_j$ is an energy scale, different quantum states evolve over time under the Hamiltonian ${\hat{H}}_{j,{{\mathcal {P}}}{{\mathcal {T}}}} ({\hat{H}}_{j,\mathcal {APT}} )$ at the same rate. In order to ensure generality, we assume that $s_j=1$ for both ${\hat{H}}_{j,{{\mathcal {P}}}{{\mathcal {T}}}}$ and ${\hat{H}}_{j,\mathcal {APT}}$. We define non-unitary operators ${\hat{U}}_{j,{{\mathcal {P}}}{{\mathcal {T}}}} = exp(-i t {\hat{H}}_{j,{{\mathcal {P}}}{{\mathcal {T}}}} )$ and ${\hat{U}}_{j,\mathcal {APT}} = exp(-i t {\hat{H}}_{j,\mathcal {APT}} )$, which can be realized in experimental setup as shown in Fig. 1, here we set $\hbar = 1$. The $\mathcal {APT}$-symmetric non-unitary operator is described as

**Figure 3:**
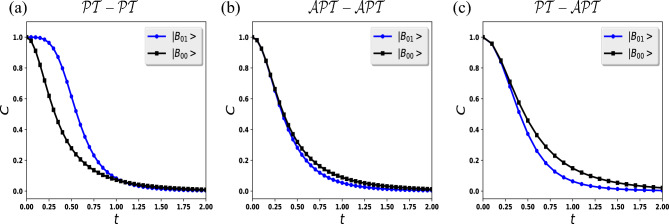
The time evolution of the concurrence in (**a**) ${{\mathcal {P}}}{{\mathcal {T}}}$-${{\mathcal {P}}}{{\mathcal {T}}}$ symmetry-broken regime, (**b**) $\mathcal {APT}$-$\mathcal {APT}$ symmetry-broken regime and (**c**) ${{\mathcal {P}}}{{\mathcal {T}}}$-$\mathcal {APT}$ symmetry-broken regimes by using different initial Bell states ${|{B_{00}}\rangle }= \frac{1}{\sqrt{2}} \left( {|{00}\rangle } + {|{11}\rangle } \right)$ and ${|{B_{01}}\rangle }= \frac{1}{\sqrt{2}} \left( {|{01}\rangle } + {|{10}\rangle } \right)$. We have taken different values of degree of non-Hermiticity (**a**) $r_1 =r_2= 1.1$ for the same ${{\mathcal {P}}}{{\mathcal {T}}}$-${{\mathcal {P}}}{{\mathcal {T}}}$ symmetry-broken regime, (**b**) $r_1 =r_2= 0.9$ for the same $\mathcal {APT}$-$\mathcal {APT}$ symmetry-broken regime and (**c**) $r_1 =1.1, r_2= 0.9$ for different ${{\mathcal {P}}}{{\mathcal {T}}}$-$\mathcal {APT}$ symmetry-broken regimes.

3 $$\begin{aligned} {\hat{U}}_{j,{{\mathcal {P}}}{{\mathcal {T}}}} = R_{HWP}(\phi _1) R_{QWP}(2 \phi _1) L\left( \eta _1,\eta _2\right)&R_{HWP}\left( -\phi _1 + \pi /4 \right) R_{QWP} \left( 0 \right) , \end{aligned}$$

and $\mathcal {APT}$-symmetric non-unitary operator is expressed as

4 $$\begin{aligned} {\hat{U}}_{j,\mathcal {APT}} = R_{QWP}(0) R_{HWP}( \pi /4) L \left( \eta _3,\eta _3 \right)&R_{QWP}\left( \theta _1 \right) R_{HWP} \left( \theta _2 \right) , \end{aligned}$$

where the loss-dependent operator can be depicted as

5 $$\begin{aligned} L\left( \eta _i,\eta _i\right) = \begin{pmatrix} 0 &{} \sin (2 \eta _i) \\ \sin (2 \eta _i) &{} 0 \end{pmatrix}. \end{aligned}$$

Loss-dependent operators can be realized by combining beam displacers (BDs) and two half-wave plates (HWPs) set at angles $\eta _i$ and $\eta _j$, for more detail please see^45^. In the above equations, $R_{HWP}$ is defined as the rotation operator of half-wave-plates (HWP)

6 $$\begin{aligned} R_{HWP} (\alpha ) = \begin{pmatrix} \cos (2\alpha ) &{} \sin (2\alpha ) \\ \sin (2\alpha ) &{} -\cos (2\alpha ) \end{pmatrix}, \end{aligned}$$

and $R_{QWP}$ is defined as a rotation operator of a quarter-wave plate (QWP),

7 $$\begin{aligned} R_{QWP} (\beta ) = \begin{pmatrix} \cos ^2(\beta )+i \sin ^2(\beta ) &{} \cos (\beta ) \sin (\beta ) (1-i) \\ \cos (\beta ) \sin (\beta ) (1-i) &{} \sin ^2(\beta )+i \cos ^2(\beta ) \end{pmatrix}. \end{aligned}$$

Using an ${{\mathcal {P}}}{{\mathcal {T}}}$-symmetric or $\mathcal {APT}$-symmetric system, we can calculate the total non-Hermitian Hamiltonian of two qubits (1, 2) as

8 $$\begin{aligned} {\hat{H}}= \left\{ \begin{array}{ccccccc} {\hat{H}}_{1,{{\mathcal {P}}}{{\mathcal {T}}}} &{} \otimes I + I \otimes {\hat{H}}_{2,{{\mathcal {P}}}{{\mathcal {T}}}} &{} &{} &{} &{} &{} (a) \\ {\hat{H}}_{1,\mathcal {APT}} &{} \otimes I + I \otimes {\hat{H}}_{2,\mathcal {APT}} &{} &{} &{} &{} &{} (b) \\ {\hat{H}}_{1,{{\mathcal {P}}}{{\mathcal {T}}}} &{} \otimes I + I \otimes {\hat{H}}_{2,\mathcal {APT}} &{} &{} &{} &{} &{} (c) \\ \end{array} \right. \end{aligned}$$

Non-unitary operator $U(t)= exp(- i {\hat{H}}t)$ for the two-qubit Hamiltonian can be expressed as follows

9 $$\begin{aligned} {\hat{U}}(t)= \left\{ \begin{array}{ccccccc } {\hat{U}}_{1,{{\mathcal {P}}}{{\mathcal {T}}}}(t) &{} \otimes &{} {\hat{U}}_{2,{{\mathcal {P}}}{{\mathcal {T}}}}(t) &{} &{} &{} &{} (a) \\ {\hat{U}}_{1,\mathcal {APT}}(t) &{} \otimes &{} {\hat{U}}_{2,\mathcal {APT}}(t) &{} &{} &{} &{} (b) \\ {\hat{U}}_{1,{{\mathcal {P}}}{{\mathcal {T}}}}(t) &{} \otimes &{} {\hat{U}}_{2,\mathcal {APT}}(t) &{} &{} &{} &{} (c) \\ \end{array} \right. \end{aligned}$$

By using the time-dependent density matrix, we can capture the nonunitary dynamics of combined systems

10 $$\begin{aligned} \rho (t)= \frac{U(t) \rho (0) U^\dagger (t) }{ \text {Tr} \big [U(t) \rho (0) U^\dagger (t) \big ] }. \end{aligned}$$

The two qubits in our proposed experiment are two photons with orthogonally polarized states ${|{H}\rangle }$ and ${|{V}\rangle }$. As shown in Fig. 1, the initial entangled Bell states of two photons are generated by a spontaneous parametric down-conversion process (left panel), then each photon undergoes an independent time evolution. In this work, we considered two different kinds of initial Bell states: ${|{B_{00}}\rangle } = \frac{1}{\sqrt{2}} \left( {|{00}\rangle } + {|{11}\rangle } \right)$ and ${|{B_{01}}\rangle }= \frac{1}{\sqrt{2}} \left( {|{01}\rangle } + {|{10}\rangle } \right)$. We can generate a relative phase between two photons by sandwich structure device (QWP-HWP-QWP) as presented in Fig. 1. In the experiment, the density matrix can be constructed at any time *t* by quantum state tomography^62^ as they pass through the time evolution section. To quantify entanglement between two photons, we calculate the concurrence^63^

11 $$\begin{aligned} C= \text {max}\big [ 0, \sqrt{\lambda _1}- \sqrt{\lambda _2}- \sqrt{\lambda _3}- \sqrt{\lambda _4} \big ], \end{aligned}$$

here, $\lambda _i (i=1,2,3,4)$ defines the eigenvalues of the evolution matrix ${\mathcal {R}}= \rho \left( \sigma _y \otimes \sigma _y \right) \rho ^* \left( \sigma _y \otimes \sigma _y \right)$ in decreasing order, where $\sigma _y$ describes the *y*-Pauli matrix.

## Identical experimental construction for both qubits

We dived this section into two parts, wherein the first part we would like to discuss the dynamics of the qubits in an identical ${{\mathcal {P}}}{{\mathcal {T}}}$-${{\mathcal {P}}}{{\mathcal {T}}}$ experimental setup, and in the second part, we emphasize on the finding of the $\mathcal {APT}$-$\mathcal {APT}$ experimental setup.

### Dynamics of qubits in ${{\mathcal {P}}}{{\mathcal {T}}}$-${{\mathcal {P}}}{{\mathcal {T}}}$ systems

As a first step, we consider the case as illustrated in Eq. (9a) in which both qubits evolve in ${{\mathcal {P}}}{{\mathcal {T}}}$-${{\mathcal {P}}}{{\mathcal {T}}}$ symmetric systems. For this, we explore the time-evolution of entanglement when the two qubits are evolving in a ${{\mathcal {P}}}{{\mathcal {T}}}$-symmetric unbroken regime $(r_1=r_2=r<1)$. Fig.  2a,c shows the evolution of entanglement when: $r=0.1$ (blue curve), (ii) $r=0.5$ (black curve), and (iii) $r=0.9$ (red curve). Here for the case Fig. 2a we take the initial Bell state ${|{B_{00}}\rangle }= \frac{1}{\sqrt{2}} \left( {|{00}\rangle } + {|{11}\rangle } \right)$ and Fig. 2c for the initial Bell state ${|{B_{01}}\rangle }= \frac{1}{\sqrt{2}} \left( {|{01}\rangle } + {|{10}\rangle } \right)$. In the case of Figs.  2a,c, we note that the concurrence has periodic oscillation with the period $T^{00}_{{{\mathcal {P}}}{{\mathcal {T}}}} = \pi /(2\sqrt{1-r^2})$ and $T^{01}_{{{\mathcal {P}}}{{\mathcal {T}}}} = \pi /(\sqrt{1-r^2})$ respectively, here for simplicity we take $r_1=r_2=r$. There is a periodic oscillation of the concurrence for initial condition ${|{B_{00}}\rangle }({|{B_{01}}\rangle })$ during time evolution, with minimal values 0.98 (0.9), 0.60 (0.22) and 0.10 (0.006) for $r=0.1$, $r=0.5$, and $r=0.9$, respectively, at different times, while the peak value for all cases equals 1. We find that the frequency of oscillations increases as parameter *r* decreases for both cases Figs. 2a,c. To some extent, both cases are identical but the concurrence decreases less for the initial Bell state ${|{B_{00}}\rangle }$ as compared to ${|{B_{01}}\rangle }$ and both Bell states have different time periods.

**Figure 4:**
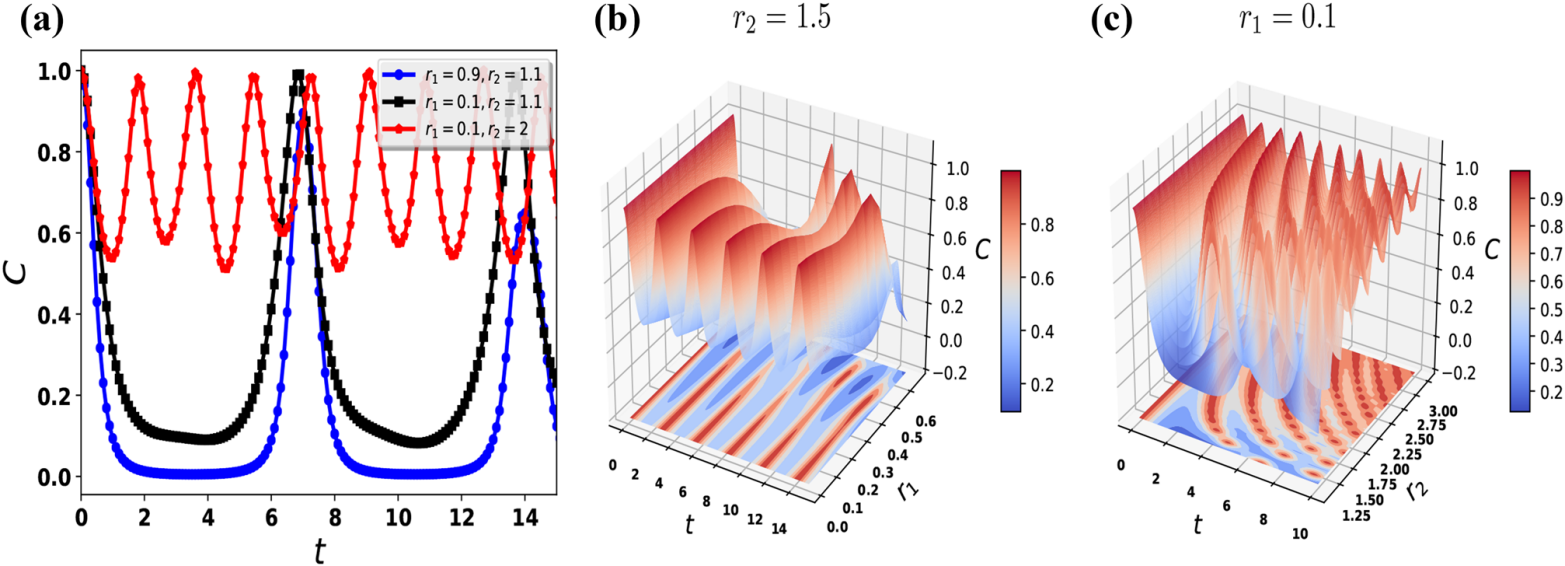
Using the Bell state ${|{B_{01}}\rangle }= \frac{1}{\sqrt{2}} \left( {|{01}\rangle } + {|{10}\rangle } \right)$ as the initial condition, we see the time evolution of the concurrence of the two qubits. (**a**) The dynamics of the concurrence in the ${{\mathcal {P}}}{{\mathcal {T}}}$ and $\mathcal {APT}$-symmetric systems. (**b**) The time evolution of concurrence in the ${{\mathcal {P}}}{{\mathcal {T}}}$ and $\mathcal {APT}$-symmetric systems when we take $r_2 = 1.5$. (**c**) The dynamics of concurrence in the ${{\mathcal {P}}}{{\mathcal {T}}}$ and $\mathcal {APT}$-symmetric systems when we consider $r_1 = 0.1$.

The above results can be illustrated by some physical explanations. We know that *r* describes the degree of non-Hermiticity of the system, so by decreasing the value of *r* we increase the non-Hermitian part $i \sigma _j$ of the Hamiltonian. This results in a shorter oscillation period since the energy of the ${{\mathcal {P}}}{{\mathcal {T}}}$-symmetric system increases accordingly. Therefore, we have preservation of entanglement for a longer period of time for small values of *r*. This entanglement preservation gets strong when we consider the Bell state ${|{B_{00}}\rangle }$ as an initial condition. Furthermore, we also investigate the dynamics of the entangled photons in the ${{\mathcal {P}}}{{\mathcal {T}}}$-symmetric broken regime $(r_1=r_2=r>1)$ for different initial Bell states ${|{B_{00}}\rangle }({|{B_{01}}\rangle })$ as presented in Fig. 3a. We note that the sudden death of the entanglement for $r_1 =r_2= 1.1$, and as we increase the value of the non-Hermitian part *r* the sudden death time decreases. We also report that the entanglement decays slower for the initial Bell state ${|{B_{01}}\rangle }$ as compared to the ${|{B_{00}}\rangle }$ as shown in Fig.  3a. At the end of this subsection, we would like to discuss a more general case, when both qubits evolve in different forming, i.e., $r_1\ne r_2$. Despite non-periodic oscillations in the concurrence, these results are not very different from previous cases, so we have not presented them here to avoid cumbersome paper length, the non-periodic oscillations appear when one qubit is in the broken-symmetry regime and the other one is in the unbroken-symmetry regime.

**Figure 5:**
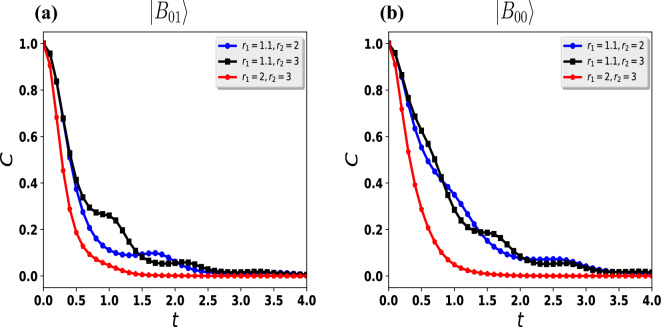
Using the Bell state (**a**) ${|{B_{01}}\rangle }$ and (**b**) ${|{B_{00}}\rangle }$ as the initial condition, we plot the time dynamics of the concurrence of the two qubits for ${{\mathcal {P}}}{{\mathcal {T}}}$ and $\mathcal {APT}$-symmetric systems in broken and unbroken regimes, respectively.

### Dynamics of qubits in $\mathcal {APT}$-$\mathcal {APT}$ systems

In the second part, we study the case as given in Eq. (9b) in which initially entangled qubits ${|{B_{00}}\rangle }({|{B_{01}}\rangle })$ evolve in identically experimental construction $\mathcal {APT}$-$\mathcal {APT}$. In an $\mathcal {APT}$-symmetric unbroken regime, we study the time-evolution of entanglement $(r_1=r_2=r>1)$, we also used $r_1=r_2=r$ here to simplify the analysis. As shown in Fig. 2b,d, entanglement evolution for $r =3$ (blue curve), (ii) $r =2$ (black curve) and (iii) $r =1.1$ (red curve), for the Fig.  2b, we begin with the initial Bell state ${|{B_{00}}\rangle }= \frac{1}{\sqrt{2}} \left( {|{00}\rangle } + {|{11}\rangle } \right)$ and for the case Fig.  2d we take the initial Bell state ${|{B_{01}}\rangle }= \frac{1}{\sqrt{2}} \left( {|{01}\rangle } + {|{10}\rangle } \right)$. Based on the following scenario, we note that the concurrence has periodic oscillation with the period $T^{00}_{\mathcal {APT}} = \pi /(2\sqrt{r^2-1})$ and $T^{01}_{\mathcal {APT}} = \pi /(\sqrt{r^2-1})$ for the Fig. 2b,d respectively. In this special scenario, we also note periodic oscillation of the entanglement for both initial conditions ${|{B_{00}}\rangle }({|{B_{01}}\rangle })$ for the identically experimental construction $\mathcal {APT}$-$\mathcal {APT}$. We notice that in both cases the frequency of the entanglement oscillations increases as the parameter *r* increases, however, at the same time, the entanglement amplitude decreases less with increasing *r*. As we already discussed in the previous part, both cases are identical however we note that the concurrence decreases less for the initial Bell state ${|{B_{00}}\rangle }$ as compared to ${|{B_{01}}\rangle }$. Thus, large values of *r* lead to the preservation of entanglement for a longer period of time for the initial Bell state ${|{B_{00}}\rangle }$. Furthermore, we examine the dynamics of entangled photons in the $\mathcal {APT}$-symmetric broken regime $(r<1)$ for two unlike initial Bell states ${|{B_{00}}\rangle }({|{B_{01}}\rangle })$ as shown in Fig. 3b. We observe, the entanglement exponentially ended for $r = 0.9$, and as we decrease the value *r* the sudden death time decreases. Similar to the case ${{\mathcal {P}}}{{\mathcal {T}}}$-${{\mathcal {P}}}{{\mathcal {T}}}$, here also our findings show that the entanglement decays slower for the initial Bell state ${|{B_{00}}\rangle }$ versus ${|{B_{01}}\rangle }$ as predicted in Fig.  3b.

## Different ${{\mathcal {P}}}{{\mathcal {T}}}$-$\mathcal {APT}$ experimental construction for both qubits

In this section, we discuss in detail, unlike experimental construction for both qubits, which is in contradistinction to the previous section. Here, we consider that the first qubit passes through the ${{\mathcal {P}}}{{\mathcal {T}}}$ experimental setup, and the second qubit moves through the $\mathcal {APT}$ experimental setup. In this regard, Fig. 4 presents the dynamical evolution of the entanglement. For the first case, we consider $r_1 =0.9$ and $r_2= 1.1$ near the exceptional point. We note that the sudden decay of entanglement and later revives is shown in Fig.  4a. In another scenario, the first qubit, we move away from the exceptional point $r_1 =0.1$, and the second qubit is still near the exceptional point $r_2= 1.1$ as shown in Fig. 4a with a black square line. We notice that the entanglement decays however do not have sudden death and complete revivals can also be seen as plotted in Fig. 4a with a black-square line. As both qubits move away from the exceptional points, we find that the entanglement does not decay but starts to oscillate with some frequencies as shown in Fig.  4a,b,c. We note that the entanglement is well preserved if we move away from the exceptional points in the unbroken regimes of the ${{\mathcal {P}}}{{\mathcal {T}}}$-symmetric system as given in Fig. 4b and for the $\mathcal {APT}$-symmetric system as shown in Fig. 4c. With this, we conclude that entanglement can be well preserved when we consider our experimental parameters quite away from the exceptional points. Theoretically, for this special case, it is hard to investigate the oscillation frequency of the entanglement as the dynamics of the entanglement get complex. Here, we consider only one initial Bell state ${|{B_{01}}\rangle }$ as we do not find much difference in the entanglement for the second initial condition ${|{B_{00}}\rangle }$. Therefore, to avoid repetition in results, we do not present them here. However, we note a delay in the sudden death of the entanglement for the initial Bell state ${|{B_{00}}\rangle }$ as compared to the Bell state ${|{B_{01}}\rangle }$ as predicted in Fig. 3c. We also calculate the dynamics of entanglement for the ${{\mathcal {P}}}{{\mathcal {T}}}$ and $\mathcal {APT}$-symmetric systems in broken and unbroken regimes, respectively as shown in Fig. 5. We notice non-periodic oscillations of entanglement as predicted in Fig.  5a,b. We note that the broken ${{\mathcal {P}}}{{\mathcal {T}}}$-symmetric regime has a strong effect as compared to the unbroken $\mathcal {APT}$-symmetric regime, therefore the entanglement decays as predicted in Fig. 5. From Fig. 5, we also study that the initial Bell state ${|{B_{00}}\rangle }$ decays fast as compared to the initial Bell state ${|{B_{01}}\rangle }$.

## Summary and conclusion

Quantum states in the $\mathcal{P}\mathcal{T}$-symmetric system, as well as the $\mathcal {APT}$-symmetric system, are evolvable by applying the non-unitary evolution operator, we examine how entangled states evolve over time. The nonunitary operator is implemented by decomposing it into unitary matrices and loss-dependent operators. We can achieve the $\mathcal{P}\mathcal{T}$-symmetric system as well as the $\mathcal {APT}$-symmetric system by linear optical elements. All nonunitary operators in the $\mathcal{P}\mathcal{T}$-symmetric systems and $\mathcal {APT}$-symmetric systems can be realized using this approach. This report explores how entanglement between two qubits evolves over time in $\mathcal{P}\mathcal{T}$-$\mathcal{P}\mathcal{T}$ symmetric system, $\mathcal {APT}$-$\mathcal {APT}$ symmetric system, and $\mathcal{P}\mathcal{T}$-$\mathcal {APT}$ symmetric system. Non-periodic oscillations, Periodic oscillations, delayed vanishing, rapid decay, and sudden death of entanglement are all observed in our theoretical simulations for different initial conditions and for various system parameters. We noted that the entanglement oscillates periodically when the non-Hermitian part $r_1=r_2=r$ is taken quite away from the exceptional point for both cases, i.e., $\mathcal{P}\mathcal{T}$-$\mathcal{P}\mathcal{T}$ symmetric and $\mathcal {APT}$-$\mathcal {APT}$ symmetric unbroken regimes. We also observed that in both cases ($\mathcal{P}\mathcal{T}$-$\mathcal{P}\mathcal{T}$ and $\mathcal {APT}$-$\mathcal {APT}$) the frequency of the entanglement oscillations increase as the parameter *r* is taken away from exceptional points, and at the same time, the entanglement amplitude decreases less. This shows that the entanglement is well-preserved for these scenarios. Nonperiodic oscillations of Entanglement were detected when both qubits evolve through in different forming, i.e., $r_1 \ne r_2$ for both cases, i.e., $\mathcal{P}\mathcal{T}$-$\mathcal{P}\mathcal{T}$ symmetric and $\mathcal {APT}$-$\mathcal {APT}$ symmetric unbroken regimes. We also noted that as we moved near the exceptional point, the entanglement decayed rapidly and later revived. However, for the $\mathcal{P}\mathcal{T}$-$\mathcal{P}\mathcal{T}$ symmetric and $\mathcal {APT}$-$\mathcal {APT}$ symmetric broken regimes, we found the entanglement of sudden death. It is really an interesting finding that the entanglement survives for a longer period of time for the Bell state ${|{B_{00}}\rangle }$ as compared to the Bell state ${|{B_{01}}\rangle }$. We also observed that the entanglement time periods $T^{00}_{\mathcal{P}\mathcal{T}} = \pi /(2\sqrt{1-r^2})$ ($T^{00}_{\mathcal {APT}} = \pi /(2\sqrt{r^2 -1})$) and $T^{01}_{\mathcal{P}\mathcal{T}} = \pi /(\sqrt{1-r^2})$($T^{01}_{\mathcal {APT}} = \pi /(\sqrt{r^2 -1})$) are different for different Bell initial conditions ${|{B_{00}}\rangle }$ and ${|{B_{01}}\rangle }$, respectively, for $\mathcal{P}\mathcal{T}(\mathcal {APT})$-symmetric unbroken regimes. When both qubits evolve different experimental construction i.e., $\mathcal{P}\mathcal{T}$-$\mathcal {APT}$ symmetric unbroken regime, we noted that the entanglement oscillates with two different oscillation frequencies and the entanglement is well protected for the case when both qubits’ non-Hermitian parts are taken quite away from the exceptional points. For this special case, the dynamics of the entanglement get complex therefore it is not possible to find a theoretical formula for the time period of entanglement oscillations. By examining the phenomena found in this scientific report, we can gain a better understanding of quantum open systems. In addition to $\mathcal{P}\mathcal{T}$-$\mathcal{P}\mathcal{T}$ symmetric systems, $\mathcal {APT}$-$\mathcal {APT}$ symmetric systems, $\mathcal{P}\mathcal{T}$-$\mathcal {APT}$ symmetric systems, and other non-Hermitian quantum systems, the present work opens up a new area for future studies in quantum entanglement dynamics in multiqubit systems.

## Data Availability

The datasets generated during and/or analyzed during the current study are available from the corresponding author upon reasonable request.
